# Characterizing Cellular Responses During Oncolytic Maraba Virus Infection

**DOI:** 10.3390/ijms20030580

**Published:** 2019-01-29

**Authors:** Golnoush Hassanzadeh, Thet Naing, Tyson Graber, Seyed Mehdi Jafarnejad, David F. Stojdl, Tommy Alain, Martin Holcik

**Affiliations:** 1 Molecular Biomedicine Program, Children′s Hospital of Eastern Ontario Research Institute, Ottawa, ON K1H 8L1, Canada; ghassanz@ualberta.ca (G.H.); ThetFatica@cunet.carleton.ca (T.N.); rnaguru@gmail.com (T.G.); dave@arc.cheo.ca (D.F.S.); tommy@arc.cheo.ca (T.A.); 2 Centre for Cancer Research and Cell Biology (CCRCB), Queen′s University Belfast, 97 Lisburn Road, Belfast BT9 7AE, UK; sm.jafarnejad@qub.ac.uk; 3 Department of Health Sciences, Carleton University, Ottawa, ON K1S 5B6, Canada

**Keywords:** translation, eIF5B, IRES, XIAP, Bcl-xL, MG-1

## Abstract

The rising demand for powerful oncolytic virotherapy agents has led to the identification of Maraba virus, one of the most potent oncolytic viruses from Rhabdoviridae family which displays high selectivity for killing malignant cells and low cytotoxicity in normal cells. Although the virus is readied to be used for clinical trials, the interactions between the virus and the host cells is still unclear. Using a newly developed interferon-sensitive mutant Maraba virus (MG1), we have identified two key regulators of global translation (4E-BP1 and eIF2α) as being involved in the regulation of protein synthesis in the infected cells. Despite the translational arrest upon viral stress, we showed an up-regulation of anti-apoptotic Bcl-xL protein that provides a survival benefit for the host cell, yet facilitates effective viral propagation. Given the fact that eIF5B canonically regulates 60S ribosome subunit end joining and is able to replace the role of eIF2 in delivering initiator tRNA to the 40S ribosome subunit upon the phosphorylation of eIF2α we have tested whether eIF5B mediates the translation of target mRNAs during MG1 infection. Our results show that the inhibition of eIF5B significantly down-regulates the level of *Bcl-xL* steady-state mRNA, thus indirectly attenuates viral propagation.

## 1. Introduction

Maraba virus has been recently characterized as an effective oncolytic vaccine for the purpose of cancer immunotherapy. It is a negative single-stranded RNA virus from *Rhabdoviridae* family with rapid replication cycle within the cytoplasm of the host cells. The standard serological tests and further phylogenetic analysis by aligning Maraba Large protein to all members of the *Rhabdoviridae* family revealed its close relationship to Vesicular Stomatitis Virus (VSV) and classified the virus as a vesiculovirus [1,2]. Owing to the similar antigenic properties between Maraba virus and VSV, a well-known oncolytic virus, the oncolytic potency and safety profile of Maraba virus have also been evaluated in recent studies [3,4]. These findings suggested that Maraba virus demonstrates selective tumor-killing activities and low cytotoxicity in normal cell lines [2,5]. In an attempt to further enhance the tumor-selective properties of Maraba virus, the equivalent mutations which were previously described to have improved the oncolytic potency of VSV were introduced into the wild-type Maraba virus. These genetic modifications were in the sequences of Matrix and Glycoprotein genes of the virus (L123W and Q242R, respectively) and have further attenuated its virulence in normal cells [2,3]. Thus, the therapeutic efficacy of this attenuated strain of Maraba virus, known as MG1, found in the pre-clinical studies had led to the world′s first clinical trial at The Ottawa Hospital. However, the exact mechanism of propagation of the virus and the host-virus interactions are still unclear.

Viruses are dependent on the cellular machinery of their host for efficient propagation. Despite carrying the components for the transcription of their genomes, all viruses rely on the translation mechanism of their host for protein synthesis [6]. Therefore, the interplay between the virus and host cells is of particular importance for both the viral protein synthesis and effective anti-viral responses. For example, the rapid inhibition of cellular global translation is known as one of the effective anti-viral strategies that represses the propagation of viruses in the infected cells. However, many viruses use an alternate mode of translation to circumvent the shut-down of global translation in their hosts [7,8].

The initiation of translation is considered a critical control point in the regulation of protein synthesis. It is therefore the key point for maintaining cellular function under physiological and pathophysiological conditions. Majority of global mRNA translation proceeds in a cap-dependent mechanism that requires binding of specific proteins termed initiation factors to the 5′ cap structure of the mRNA [9,10,11]. During various cellular stresses, two major translation initiation complexes, eIF4F (consisting of eIF4E, eIF4A and eIF4G) and the ternary complex (consisting of eIF2, GTP and Met-tRNAi), are targeted by distinct signaling processes for the regulation of translation [11,12,13,14]. Previous studies have shown that during some viral infections—for example, Encephalomyocarditis virus (EMCV) or VSV—the formation of the eIF4F complex is prevented through the conformational changes in eIF4E *via* binding of the 4E-binding protein 1 (4E-BP1), leading to the translation inhibition [10,15]. Furthermore, the assembly of 43S pre-initiation complex, composed of the ternary complex, 40S small ribosomal subunit and eIF3 is affected in response to the infection with certain viruses [14].

Eukaryotic Initiation Factor 2 (eIF2) is one of the essential components of the ternary complex responsible for the delivery of the initiator tRNA, Met-tRNA, to the P site of the small ribosomal subunit in a GTP-dependent manner [16,17,18]. During cellular stress, phosphorylation of α subunit of eIF2 leads to the formation of an inactive eIF2-GDP-eIF2B complex that blocks GDP-to-GTP recycling. This limits the number of available active eIF2 proteins for the assembly of the ternary complex and 43S resulting in the inhibition of the global translation initiation [19,20]. Among the identified serine-threonine kinases with roles in the phosphorylation of eIF2α in response to distinct types of cellular stress, the RNA-dependent protein kinase R (PKR) is proposed to become activated following the recognition of double-stranded RNA during viral infections [16,21]. Some studies have linked the activation of PKR and further phosphorylation of eIF2α with the formation of stress granules in the infected cells [22]. It was suggested that the assembly of stress granules occurs upon the depletion of the small ribosomal subunit from the active ternary complex [23,24]. This event subsequently provides a coping strategy for the stressed cells to restrict viral propagation and promote their chance of survival. As a result, many viruses have evolved mechanisms to evade the sequestration of their mRNA in the stress granules by blocking their formation [25,26,27]. In addition, replacing the role of eIF2α in Met-tRNA delivery with other translation factors such as eIF5B has been suggested as a backup strategy through which some viruses and stressed cells promote the translation of viral and select cellular mRNAs upon the phosphorylation of eIF2α. eIF5B is the eukaryotic ortholog of bacterial IF2 that mediates the translation elongation step by catalyzing 60S ribosomal subunit joining [28,29,30]. However, recent studies suggested a non-canonical function for eIF5B in the delivery of the initiator tRNA under certain types of cellular stresses, such as HCV and CSFV infection and serum-starvation [31,32,33].

Despite the translational arrest during cellular stress, a substantial body of evidence shows that an alternate mode of translation, termed cap-independent translation, is used by the cells to promote the synthesis of mRNAs involved in the cell survival or apoptosis pathways [34]. Many of these mRNAs harbor an internal ribosome entry site (IRES) within their 5′ untranslated region which is believed to facilitate the recruitment of the ribosome when the cap-dependent mechanism of translation is inhibited [34,35].

In the present study, we investigated the regulation of global and mRNA-specific translation during Maraba MG1 infection. We demonstrate the inhibition of host′s cytoplasmic translation during MG1 infection and the involvement of eIF2α phosphorylation in the repression of both host cytoplasmic translation and viral protein synthesis. Using ribosomal profiling we assessed the cellular mRNAs that are being selectively translated during Maraba virus infection. Our data suggested that a distinct cohort of IRES-containing mRNAs with roles in the apoptosis pathways undergo selective translation and we validated these findings by focusing on Bcl-xL and XIAP, which were previously shown to be translationally co-regulated *via* IRES-dependent mechanism under certain stress conditions [36,37,38]. Finally, we explored whether selective translation of *Bcl-xL* and *MG1* mRNAs are mediated through eIF5B-dependent pathways and identified an indirect role for eIF5B in limiting the efficient replication of the virus within the host. Our data shed light on the translational control during MG1 infection which can be helpful in designing more effective oncolytic strategies in the future.

## 2. Results

### 2.1. Ribosome Profiling of MG1-Infected U343 Cells Highlights Cellular Targets of eIF2α Phosphorylation and Apoptosis

**Figure 1:**
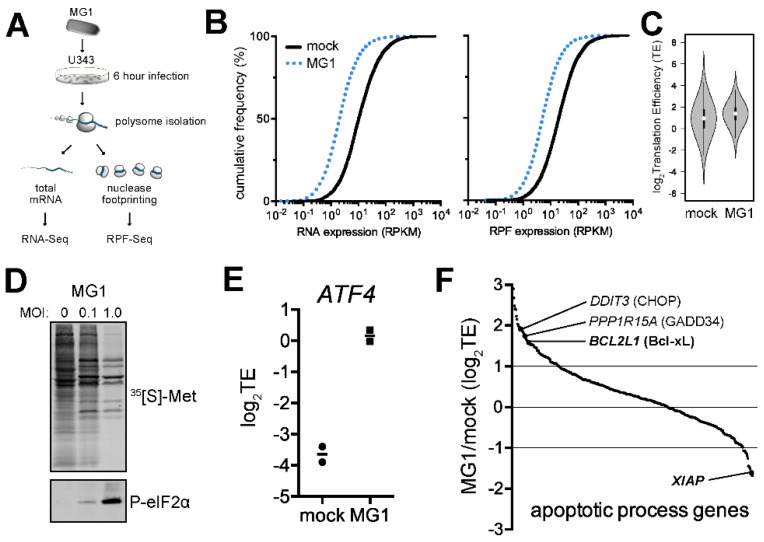
Ribosome profiling of MG1-infected U343 cells highlights cellular targets of eIF2α phosphorylation and apoptosis. (**A**) Schematic of the ribosome profiling strategy used. Polysomes were fixed using cycloheximide prior to extraction. RPF; Ribosome protected fragments. (**B**) Cumulative frequency distributions of gene expression at the transcriptome (RNA) and translatome (RPF) levels in mock and MG1-infected cells. (**C**) Violin plots showing distribution of the translation efficiency (TE = RPF/RNA) for sequenced genes between mock and MG1-infected U343 cells. The median (central dot), interquartile range (black box), 95% confidence interval (vertical line) are shown, while the grey area represents a density plot wherein the width is proportional to the frequency of the TE values. (**D**) Radiograph (top) showing nascent peptide synthesis in mock-infected U343 cells (0 MOI) or increasing MOI of MG1 virus. Western blot (bottom) probing for phospho-eIF2α. (**E**) TE of *ATF4*, a known translational target of phospho-eIF2α, in mock- vs. MG1-infected U343 cells. (**F**) Distribution of TE for genes classified as “apoptotic processes” by the Gene Ontology consortium. Two known translation substrates of phospho-eIF2α, CHOP and GADD34 demonstrated enhanced TE, while other apoptotic regulators such as Bcl-xL and XIAP exhibited antipodal TEs. **** *p* < 0.0001; determined using a non-parametric Kolmogorov-Smirnov statistical test.

Recent studies demonstrated an abrupt shut-down of cap-dependent translation during VSV infection [7]. Due to the close similarity between Maraba virus and VSV, we hypothesized that MG1 infection might lead to similar suppression of the global translation in the host cells. To determine whether alternative mechanisms of translational regulation play a role in regulating the cellular response to infection, we profiled the translated set of genes (i.e., the translatome) in MG1-infected U343 cells using ribosome profiling (Figure 1A) [39]. Following 6 h of mock- or MG1-infection of U343 cells, polysomes were stabilized with cycloheximide and the lysate was split into parallel workflows to isolate mRNA associated with ribosomes (ribosome protected fragments, RPF) and total poly(A)+ mRNA (RNA). Both mRNA populations were converted to cDNA libraries and subjected to massively parallel sequencing. Expression of both cellular mRNAs and RPFs was found to significantly decrease upon MG1 infection at 1 MOI (Figure 1B). Despite this, we observed a significant increase in the translation efficiency (TE; RPF/RNA) of a subset of cellular mRNAs 6 h post-infection at this MOI (Figure 1C). Labelling of nascent peptide chains indicated no clear change in global cellular mRNA translation at 0.1 MOI but a coincident increase in viral protein synthesis and phosphorylation of eIF2α was observed (Figure 1D). Higher MOI (1.0) led to a drastic reduction in global protein synthesis at 12 h post-infection and induced high levels of P-eIF2α (Figure 1D). At 1 MOI, ribosome profiling was able to resolve a significant de-repression in the translation of *ATF4*; this is a well-known consequence of the activation of the Integrated Stress Response (ISR) that is mediated through a phospho-eIF2α- and uORF-dependent mechanism (Figure 1E, [40]). Notably, “apoptotic process genes” that are also regulated through uORF-mediated translation such as the human orthologues of *CHOP* and *GADD34,* were also found to have increased TE during MG1 infection. Interestingly, we also found two genes that are known to be regulated by IRES-mediated translation. Unexpectedly, these 2 genes (*BCLxL* and *XIAP*) have opposite behaviors with respect to their differential TE, with *XIAP* being poorly translated and *BCLxL* being highly translated during MG1 infection. Thus, MG1 infection of U343 cells leads to dramatic changes at the level of translation, whose targeted substrates suggest the induction of the ISR and reprogramming of cellular translation.

### 2.2. MG1 Regulates Selective Translation of Viral mRNA and Bcl-xL Upon the Inhibition of Global Translation

**Figure 2:**
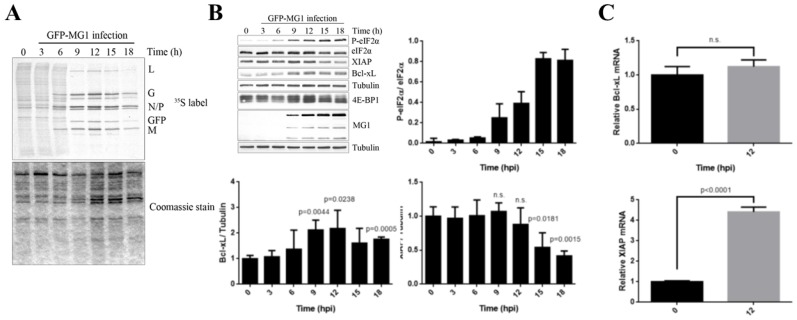
The impact of MG1 infection on the host protein synthesis. (**A**) U2OS cells were infected with MG1 at MOI = 0.1 for 18-h time course. The cells were pulsed labeled with [^35^S]-methionine for 20 min. Next, the obtained lysates were separated through 10% SDS-PAGE electrophoresis. Identity of viral proteins is indicated to the right of the image; Coomassie blue staining demonstrates the protein loading. (**B**) Western blot of U2OS cells infected with MG1 at MOI = 0.1 during an 18-h time course demonstrated phosphorylation of eIF2α, de-phosphorylation of 4E-BP1 and up-regulation of Bcl-xL protein. The levels of Bcl-xL and XIAP proteins were quantified relative to Tubulin used as a loading control. (**C**) Steady-state levels of *Bcl-xL* and *XIAP* mRNAs were determined in mock- and MG1-infected cells at 12 hpi. Ct values were normalized by the standard curve of each primer and represented relative to the geometric mean of RPL13A and RPL36. (n.s. = not significant)

To corroborate ribosome profiling data we performed [^35^S]-methionine labeling in U2OS cell infected with MG1 virus at MOI of 0.1 over the course of 18 h. Labelled proteins were subsequently separated by SDS-PAGE and detected by exposure to film. Similarly to what we observed in U343 cells we detected significant repression of cytoplasmic translation and selective synthesis of MG1 proteins starting at 6 h post infection (hpi) (Figure 2A).

To explore the possible involvement of eIF2 and 4EBP1 pathways in the translation inhibition during MG1 infection, the phosphorylation status of eIF2α and 4E-BP1 were monitored by western blot analysis. We detected the gradual phosphorylation of eIF2α and de-phosphorylation of 4E-BP1 during the time-course of MG1 infection (Figure 2B), coinciding with the onset of inhibition of host protein synthesis, suggesting that both regulatory proteins play a role in the repression of translation in MG1-infected cells. Western blot analysis of viral proteins from the same lysates confirmed the selective synthesis of MG1 proteins (Figure 2B). In addition, we examined the expression levels of Bcl-xL and XIAP in order to validate the ribosomal profiling data. Interestingly, our data confirmed selective up-regulation of Bcl-xL protein. In contrast, the levels of XIAP protein remained unchanged during the 12 hpi, after which there was a significant reduction at approximately 15 hpi (Figure 2B). To further determine whether the observed changes in Bcl-xL and XIAP protein levels are accompanied by changes in their respective mRNAs, we performed quantitative RT-PCR to examine the levels of steady-state *Bcl-xL* and *XIAP* mRNAs at 12 hpi, the time point that the maximum levels of both proteins were observed (Figure 2C). We noted that the levels of *Bcl-xL* mRNA remained unchanged, suggesting that the observed increase of Bcl-xL protein is mediated at the level of translation, thus confirming the ribosome profiling data. In contrast, we observed elevated level of steady-state *XIAP* mRNA in MG1-infected cells despite declining levels of the XIAP protein (Figure 2C).

### 2.3. Phosphorylation of eIF2α Modulates the Repression of Host’s Protein Synthesis and MG1 Propagation

**Figure 3:**
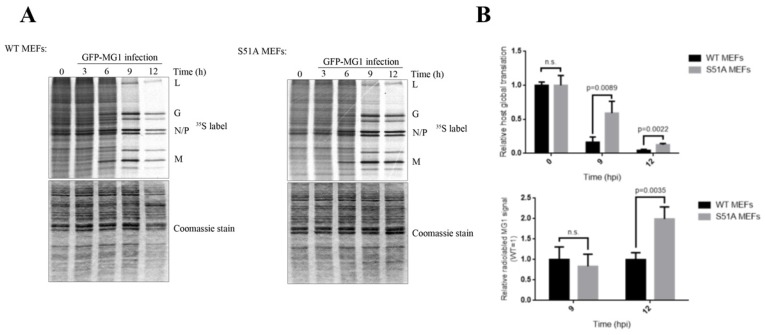
The effect of eIF2α phosphorylation on the rate of host and MG1 protein synthesis. (**A**) WT and S51A MEFs were infected with MG1 at MOI = 0.1 for the indicated time points and pulse labeled with [^35^S]-methionine for 20 min. Protein lysate were then separated through 10% SDS-PAGE gel. Coomassie blue stain was used as loading control. (**B**) Top: Densitometry of the relative radioactive signal of the area between the viral L and G bands was normalized to the entire related lane on Coomassie gel at 0, 9 and 12 hpi to determine the rate of host global translation. Bottom: the relative radioactive signal from the viral M protein to Coomassie stain was normalized to the signals from the WT MEFs at each time points to measure the rate of viral translation between the two cell lines.

**Figure 4:**
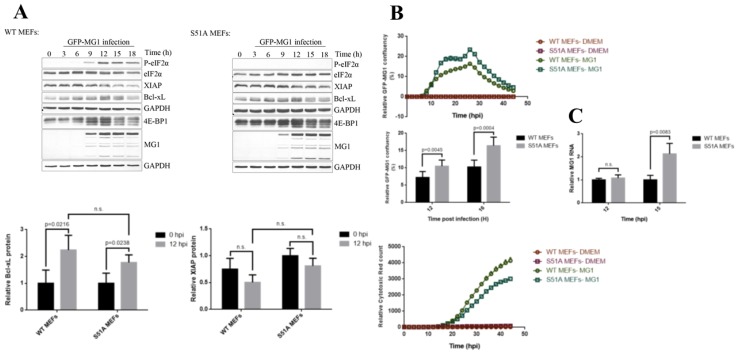
The levels of MG1 proteins and up-regulation of Bcl-xL protein are independent of eIF2α phosphorylation. (**A**) Western blot analysis from the time course infection of WT and S51A MEFs with MG1 at MOI = 0.1 demonstrates the levels of Bcl-xL, XIAP proteins and de-phosphorylation of 4E-BP1. GAPDH is shown as the loading control. Relative Bcl-xL and XIAP expression levels to GAPDH at 12 hpi were normalized to uninfected cells (Bottom). (**B**) Top: IncuCyte live cell imaging demonstrates the confluency of GFP signal in WT and S51A MEFs in real time. Y axis depicts the percentage of GFP-MG1 infected cells relative to total cell confluency. Middle: Fold GFP-MG1 confluency between the two cell lines was quantified at 12 and 16 hpi. Bottom: Cytotox Red reagent was used in cells mock-infected or infected with MG1 virus and the activity of the fluorescent red dye was monitored over a time course. Y axis represents the number of cells emitting fluorescent red (dead cells) relative to the cell confluency. (**C**) *MG1* genomic RNA was measured relative to the geometric mean of *RPL13A* and *PPIA* mRNAs in WT and S51A MEFs at 12 and 15 hpi.

Our data so far implicated eIF2 in mediating the repression of host translation in MG1-infected cells while allowing MG1 protein synthesis. To further explore the effect of eIF2α phosphorylation on the repression of host translation and selective protein synthesis of MG1 proteins, we performed [^35^S]-methionine labeling in phosphor-null eIF2α (S51A) immortalized MEFs and the genetically-paired wild type MEFs following a time-course of infection with MG1 virus at MOI of 0.1 (Figure 3). We observed that the inability of cells to phosphorylate eIF2α resulted in a marked delay in the inhibition of host translation when compared to WT cells. Interestingly, this also led to an enhanced expression of viral proteins at 12 hpi, suggesting that phosphorylation of eIF2α may be a limiting factor for both the host and viral translation. Next, we compared the expression of Bcl-xL and XIAP proteins in the aforementioned MEFs by western blot analysis. We observed that eIF2α phosphorylation status had no noticeable impact on the expression of Bcl-xL or XIAP (Figure 4A).

In order to gain a better understanding of the role of eIF2α in MG1 infection, we have compared the levels of MG1 genomic RNA between the two MEF cell lines at 12 and 15 hpi. Interestingly, we observed a marked increase in the levels of MG1 genomic RNA in the phosphor-null MEFs compared to the WT MEFs at 15 hpi (Figure 4C). This observation along with the metabolic labeling assay data suggest the negative role that eIF2α phosphorylation plays during MG1 infection in the host cells. This notion was further confirmed by monitoring the rate of viral propagation and cytotoxicity of GFP-expressing MG1 virus through live-cell imaging. Consistent with our previous observations, MG1 virus showed higher rate of infection in the S51A MEFs when compared to the WT MEFs (Figure 4B).

### 2.4. MG1-Induced Stress Granules Do Not Restrain Viral Propagation

**Figure 5:**
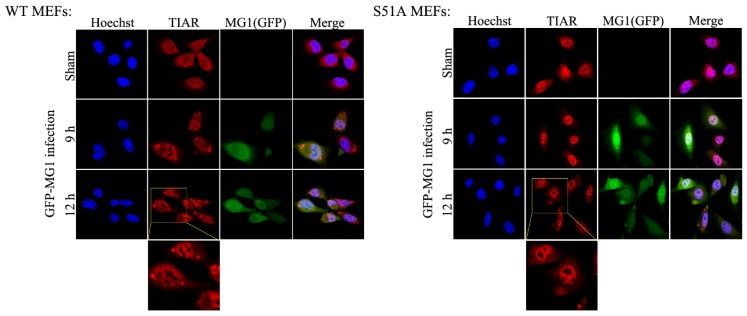
Formation of stress granules during MG1 infection does not repress viral propagation. WT and S51A MEF cell lines were infected with MG1 virus at MOI = 0.1 for a time course of 12 h as indicated. Immunofluorescence was performed to visualize the stress granules using TIAR anti-rabbit antibody. Cells were imaged by confocal microscopy using 60× water objective. Nuclei (blue) were stained by Hoechst through DAPI filter, GFP-MG1 (green) and TIAR (red) were visualized through FITC and TRITC filters respectively.

The formation of stress granules is triggered during the infection of cells with certain viruses in order to restrict viral propagation and promote cell survival [22]. Since the assembly of stress granules is closely linked to the phosphorylation of eIF2α [24,41], we decided to examine the effect of SG formation on MG1 infection. Immunofluorescence staining was performed in S51A and WT MEFs which were grown on cover-slips for 24 h and subsequently infected with 0.1 MOI of GFP-expressing MG1. We selected to use TIAR protein as a stress granule marker since it was reportedly associated with stress granule in the VSV-infected cells [42]. We observed that infection with MG1 resulted in the formation of stress granules in WT but not in S51A MEFs (Figure 5). This observation confirmed that the phosphorylation status of eIF2α is the main contributor to the formation of SGs during viral infection. In addition our data show that MG1 virus does not block the assembly of SG.

### 2.5. eIF5B Contributes to the Expression of Bcl-xL and MG1 during Viral Infection

**Figure 6:**
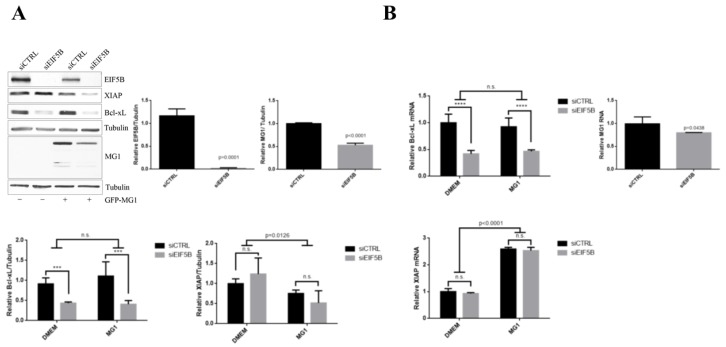
eIF5B knock-down negatively regulates the expression of Bcl-xL and MG1. (**A**) U2OS cells were transfected with eIF5B-targeting or non-targeting control siRNAs followed by 12 h of mock- or infection with MG1 at MOI = 0.1. Western blot demonstrates the level of eIF5B, Bcl-xL, XIAP, MG1 and Tubulin (loading control) proteins. The expressions of Bcl-xL and XIAP proteins were quantified relative to Tubulin by densitometry (*** *p* = 0.0009; ns, non-significant). (**B**) Steady-state levels of *Bcl-xL*, *XIAP* mRNA and MG1 genomic RNA were measured by RT-qPCR in siCTRL or siEIF5B transfected cells after 12 hpi with MG1 or DMEM treatment. Ct values were normalized by the standard curve method and represented relative to the geometric mean of RPL13 A and RPL36A (**** *p* < 0.0001; ns, non-significant).

Recent studies suggested an alternate role for eIF5B in delivering Met-tRNA upon the inactivation of eIF2 under certain cellular stress [31,33]. Moreover, we have previously demonstrated the non-canonical function of eIF5B in cap-independent translation of XIAP during serum starvation [32]. Given that we have observed enhanced translation of MG1 proteins and Bcl-xL despite increased phosphorylation of eIF2α during MG1 infection, we wished to examine the potential role of eIF5B in this process. We used western blot analysis and quantitative RT-PCR to assess the level of Bcl-xL and XIAP proteins and steady-state level of their mRNAs, as well as MG1 proteins and genomic RNA in U2OS cells transfected with siRNA targeting eIF5B and non-targeting siRNA as the negative control. Following 60 h of transfection, cells were infected with MG1 at MOI of 0.1 for 12 h. We observed reduced expression of *Bcl-xL* mRNA and protein in both uninfected and MG1-infected cells with reduced eIF5B levels (Figure 6). These data suggest that the enhanced expression of Bcl-xL observed in MG1-infected cells is not mediated by an alternative, eIF5B-dependent bypass of eIF2α phosphorylation. In contrast, although the *XIAP* mRNA levels remained unchanged, the XIAP protein levels were further decreased in MG1-infected cells with reduced eIF5B levels (Figure 6), consistent with the previously described role of eIF5B in XIAP translation during stress [32].

**Figure 7:**
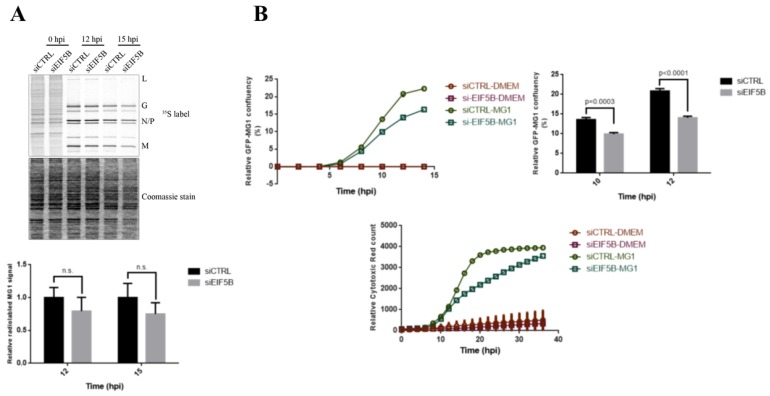
eIF5B knock-down affects the rate of MG1 infection and cytotoxicity. (**A**) Top: western blot from [^35^S]-methionine labeling translational assay, U2OS cells were transfected with siCTRL or siEIF5B, followed by MG1 infection at MOI = 0.1 for 12 and 15 h. Bottom: Densitometry of M protein signal relative to Coomassie blue stain demonstrates the rate of *MG1* mRNA translation at the indicated time points (**B**) Top left panel: U2OS cells were treated as in (**A**) and the rate of MG1 infection was monitored using IncuCyte Zoom live cell imaging. Y axis represents the percentage of GFP-MG1 infected cell relative to cell confluency. Top right panel: Fold confluency of GFP-MG1 signal at 10 and 12 hpi. Bottom panel: MG1 cytotoxicity was compared between the previously described conditions by monitoring signals from Cytotox Red reagent using IncuCyte Zoom. Y axis represents the number of cells emitting fluorescent red relative to cell confluency.

siRNA-mediated reduction of eIF5B levels resulted in approximately 20% reduction in the abundance of MG1 genomic RNA and approximately 50% reduction in the expression of MG1-encoded proteins (Figure 6). We further explored the role of eIF5B in the control of *MG1* mRNA translation by performing [^35^S]-methionine labeling in sieIF5B and siCTRL transfected cells after 12 and 15 h of MG1 infection. Our data demonstrated that reducing the level of eIF5B does not affect the rate of *MG1* mRNA translation (Figure 7A). To test the impact of eIF5B knock-down on cell viability and the rate of MG1 infection, we used the live-cell imaging to monitor the rate of cell survival and MG1 infection in the eIF5B-depleted and control cells during the viral infection. We observed that reducing the level of eIF5B significantly limits the ability of MG1 virus to propagate in the host cells and consequently enhances the cell survival during MG1 infection (Figure 7B).

## 3. Discussion

Rhabdovirus Maraba has been described as a promising oncolytic virus due to its tumor-specific activity and ability to enhance adaptive immunity [5]. Given the close relativeness of Maraba virus with VSV with respect to their amino acid sequences and antigenic properties, it is expected that both viruses demonstrate comparable OV properties [2,3]. Therefore, we speculated that Maraba virus may use similar mechanisms as VSV to propagate within the tumor cells.

Previous studies on the mechanisms of translational regulation during VSV infection proposed the involvement of eIF2α phosphorylation and de-phosphorylation of eIF4E and 4E-BP1 as main regulators of global translation in the host cells [7,43]. In agreement with these reports our demonstrated both phosphorylation of eIF2α and de-phosphorylation of 4E-BP1 during MG1 infection. Moreover, our follow-up experiments in WT and S51A MEFs highlighted the prominent role of eIF2α in controlling translation mechanism during MG1 infection. Importantly, we observed lower rate of MG1 translation at later time points in WT MEFs in comparison with S51A MEFs. Although this event may be attributed to the direct impact of eIF2α inactivation on MG1 protein synthesis, the result from the survival assay suggested that the lower rate of infection at 12 hpi in WT MEFs can be explained by the greater level of cytotoxicity, resulting in the lower chance for the virus to propagate inside the cells. Despite the significant rate of translation inhibition in WT MEFs compared to S51A MEFs at 9 and 12 hpi observed in [^35^S]-Methionine labeling experiment, we found a noticeable repression of protein synthesis after 9 hpi in the mutant MEFs suggesting the involvement of other mechanism(s) such as eIF4E-mediated pathways in the inhibition of translation. 

The data from ribosomal profiling on MG1 infected U343 glioblastoma cells suggested that a cohort of specific apotptosi-related mRNA candidates were differentially translated in the infected cells. One of the hits from the translatome analysis was *Bcl-xL* mRNA exhibiting enhanced translational efficiency during the infection. Previous studies showed that many anti-apoptotic proteins are up-regulated via IRES-mediated translation during cellular stress. Among them, Bcl-xL and XIAP have been reported to be co-regulated in a number of conditions in order to control both intrinsic and extrinsic pathways of apoptosis [36,44]. Bcl-xL, encoded by *bcl-x* gene, is an anti-apoptotic protein from the Bcl-2 family [45]. The expression of Bcl-xL is controlled at different levels of transcription, alternative splicing and translation. It has been demonstrated that *Bcl-xL* mRNA harbors a long IRES element in its 5′ UTR facilitating its selective expression during cellular stress [37,38]. The main function of Bcl-xL is the repression of the intrinsic pathway of apoptosis by preventing the oligomerization of pro-apoptotic protein, Bak, in the mitochondrial outer membrane, thus maintaining the mitochondrial membrane integrity under cellular stress conditions [45,46]. Similarly, X-linked inhibitor of apoptosis protein (XIAP, also known as BIRC4) is an anti-apoptotic protein from IAP family which inhibits caspase-3, -7 and -9 through the direct binding of its BIR (Baculoviral IAP Repeats) domains with caspases. Thus, it is able to block both the intrinsic and extrinsic pathways of apoptosis [47,48,49,50]. Our results from the time course experiment in U2OS cells confirmed the elevated expression of Bcl-xL at a translational level, however, the level of XIAP protein was decreased at later time points during MG1 infection. RT-qPCR was also performed to determine if MG1 infection had any effect on the transcription of *Bcl-xL* and *XIAP*. We observed that the expression of *Bcl-xL* mRNA remained unaffected, however, we observed a significant increase in *XIAP* steady-state mRNA level at 12 hpi. Our observations suggested that expressions of Bcl-xL and XIAP are in fact differentially regulated during MG1 infection. This is in contrast to previous reports showing frequent co-regulation of Bcl-xL and XIAP under cellular stress conditions [36,37,38]. It needs to be noted, however, that in the previously published data the stress was not evoked by viral infection and this is the first report to examine expression of these two genes during Maraba infection. Further analysis of the precise molecular mechanisms regulating Bcl-xL and XIAP expression during Maraba infection is clearly indicated. It has been previously reported that selective translation of *Bcl-xL* and/or *Bcl-2* mRNAs not only prevent the premature death of infected cells but can increase the rate of virus production. Therefore, some DNA viruses such as herpesviruses and poxviruses have evolved mechanisms to either encode proteins that mimic anti-apoptotic functions of Bcl-2 and Bcl-xL or use alternate strategies to up-regulate the expression of these proteins [51].

The abundance of available ternary complex is a critical factor for the regulation of translation initiation. Some mRNAs, however, rely on an alternative mechanism for Met-tRNA delivery to circumvent the translation initiation inhibition following eIF2α phosphorylation. Interestingly, previous studies demonstrated that during certain forms of cellular stress, the translation of IRES-harboring mRNAs such as *Bcl-xL* (during hypertonic stress), *c-Src* and *c-Myc* (during ER stress) are increased upon the inactivation of eIF2α [37,52,53]. One proposed model of eIF2-indepenent translation initiation has been observed during HCV and CSFV infection. Both viruses utilize eIF5B for the delivery of Met-tRNA into P site of 43S ribosome once eIF2 becomes inactive in the infected-cells [33,54]. Similar mechanism of eIF5B-mediated translation was described for *XIAP* mRNA through IRES-mediated translation of its long splice variant [32]. Our data showed significant down-regulation of *Bcl-xL* mRNA and consequently protein level in eIF5B depleted cells under both normal and viral stress conditions. Since eIF5B has a translation-specific role, we speculate that eIF5B knock down may negatively affect the level of *Bcl-xL* mRNAs incorporated in the translation machinery, thereby leading to their rapid degradation. We also addressed the role of eIF5B in mediating the translation of MG1 virus. Our results revealed that knocking-down eIF5B had significantly reduced the level of *MG1* RNA. However, this effect was found to be more significant at the protein level. Metabolic labeling in siCTRL and siEIF5B treated cells during a time course of MG1 infection showed that eIF5B did not affect the translation rate of MG1 viral protein, suggesting that eIF5B plays an indirect role in both translation and replication of the virus. In addition, IncuCyte live cell imaging of eIF5B-depleted cells showed higher survival rate in response to MG1 infection despite the down-regulation of Bcl-xL expression. This event may be associated with their lower rate of infection observed once levels of eIF5B are reduced in the cells. Further experiments will be required to fully understand the underlying mechanism(s) of eIF5B controlling MG1 propagation and understand the correlation between the level of available Bcl-xL protein and the rate of MG1 infection.

Overall, we have shown eIF2α phosphorylation and de-phosphorylation of 4E-BP1 contribute to the inhibition of protein synthesis in MG1-infected cells. We also observed a selective up-regulation of *Bcl-xL* mRNA translation during MG1 infection which was regulated independently of XIAP protein expression. Finally, we have identified a potential role for eIF5B in modulating MG1 propagation and Bcl-xL expression found to be mediated at transcriptional or post-transcriptional levels in eIF5B-depleted cells. In conclusion, this study was the initial step to unravel the strategies used by MG1 to reprogram the host translation machinery and its mechanism of propagation.

## 4. Materials and Methods

### 4.1. Cell Culture, Expression Constructs and Transfection

U2OS cells were grown at 37 °C in 5% CO_2_ in HyClone^TM^ High-Glucose Dulbecco’s Modified Eagle’s Medium (Thermo Scientific, Waltham, MA, USA) supplemented with 1% l-Glutamine, 100,000 U/L Penicillin, 100 μg/L Streptomycin and 10% Heat-inactivated Fetal Bovine Serum. Mouse embryonic fibroblasts (MEFs), phosphor-null eIF2α (S51A) and WT MEFs, were a gift from Dr. Maria Hatzoglou (Departments of Nutrition, Pathology and Biochemistry, Case Western University School of Medicine, Cleveland, OH, USA) and were also cultured in standard condition in serum-, antibiotic- and l-Glutamine- supplemented DMEM at 37 °C in 5% CO_2_.

Transient knock-down experiments were performed using RNAiMax reagent (Invitrogen, Burlington, ON, Canada) as per manufacturer’s protocol, siRNA targeting EIF5B (Stealth RNAi™ siRNAs, Cat#1299001, Invitrogen) and negative control siRNA (Qiagen, Cat #102720, Toronto, ON, Canada). 1.0E05 U2OS cells were seeded in 6-well plates and grown in antibiotic-free DMEM for 24 h. Cells were transfected with 30nM siEIF5B and non-targeting control siRNA for 72 h.

For MG1 infection experiments, GFP-fused interferon-sensitive mutant Maraba virus (MG1) described previously was used [2]. After 48 to 60 h of siRNA transfection, the media was removed from the cells and replaced by 2 mL complete DMEM with or without MG1 virus at MOI of 0.1 for at least 12 h. For the time-course experiments, 2.5E05 cells were seeded in 6-well plates, culture medium was replaced by complete DMEM containing MG1 virus at MOI = 0.1 and plates were incubated at 37 °C in 5% CO_2_ for 0–18 h infection. Cells were harvested for protein and RNA extractions every 3-h.

### 4.2. Ribosome Profiling

The human glioblastoma cell line U343 was obtained from the American Type Culture Collection and cultured in DMEM plus 10% FCS. For ribosome profiling, two 15-cm plates of U343 at ~80% confluence were mock-infected or infected with MG1 at a MOI of 1 and processed 6 h post-infection. For metabolic [35S]-labelling, U343 cells in a 12 well plate were infected with MG1 at a MOI of 0, 0.1 and 1. The cells were labeled for 30 min at 37 °C, 5% CO2 with complete growth media containing EasyTagTM Express Protein Labeling Mix ([^35^S]-l-methionine and [^35^S]-l-cysteine) (PerkinElmer) at 10 µCi/mL. Cells were then lysed at 12 h post-infection in radioimmunoprecipitation assay (RIPA) buffer (150 mM NaCl, 1.0% IGEPAL-CA-630, 0.5% sodium deoxycholate, 0.1% SDS, 50 mM Tris, pH 8.0). Protein lysates were separated on SDS-PAGE and transferred to a nitrocellulose membrane, followed by exposure to autoradiography film. Ribosome profiling was performed as previously described on 2 biological replicates [39]. Briefly, polysomes in U343 lysates were stabilized with 100 µg/mL cycloheximide (Bioshop, Burlington, ON, Canada) for 5 min and split into two parallel workflows. For RNA-Seq, 150 µg of total RNA was used to purify Poly-(A)+ mRNA, followed by alkaline fragmentation and 35–50 nucleotide fragments were selected. For RPF-Seq, the polysome lysate was subjected to RNase I treatment at room temperature for 45 min, followed by monosome isolation through a sucrose gradient. Purified RNA was size-selected (corresponding to 28–32 nt fragments). RNA fragments were used to create cDNA libraries as previously described [39]. Ribosomal RNA (rRNA) contamination was reduced by subtractive hybridization using biotinylated oligos that were reverse complements of abundant rRNAs. The mRNA and ribosome-footprint libraries were then amplified by PCR (10 cycles) using indexed primers and sequenced on the Illumina HiSeq 2000 platform (San Diego, CA, USA) with read length of 50 nucleotides at the McGill University and Génome Québec Innovation Centre.

### 4.3. Mapping and Analysis of Ribosome Profiling Data

The adapter sequence was removed from reads using FASTX [55] and reads that mapped to rDNA sequence by Bowtie 2 [56] were discarded. Reads were then mapped to the mouse genome (mm10) using Bowtie 2. Uniquely mapped reads with MAPQ score ≥10 were used for further analysis. For gene expression analysis, reads mapping to the coding region of RefSeq transcripts were used to calculate Reads Per Kilobase per Million total uniquely mapped reads (RPKM). Gene-level RPKMs were obtained by conflating and averaging transcript RPKMs. Genes that showed no expression (0 RPKM) at either the transcription or translation levels in either the mock or infected samples were omitted from further analysis. Translation efficiency was defined by the log_2_ ratio of RPF to total RNA RPKMs. Violin plot was created with BoxPlotR (http://shiny.chemgrid.org/boxplotr/).

### 4.4. Protein Extraction and Western Blot Analysis

For protein extraction, cells were washed twice with cold Phosphate-buffered saline (PBS), then lysed in 1× RIPA buffer (50 mM Tris-CL pH 7.4, 150 mM NaCl, 1 mM EDTA, 0.5% Sodium deoxycholate, 1% (*v*/*v*) NP-40, 0.5% (*w*/*v*) SDS) supplemented with Halt^TM^ Protease and Phosphatase Inhibitor Cocktail (Thermo Scientific #78440, Waltham, MA, USA). Bradford protein assay (Bio-Rad) was performed to measure protein concentration. Equal amount of proteins were diluted in 2× Laemmli buffer with the addition of 5% Dithiothreitol (DTT) and loaded into 10% SDS-PAGE. Protein samples were separated at the constant voltage of 150 V for 1.5 h and transferred onto the 0.2 µm PVDF membrane and the levels of following proteins were determined: rabbit anti-Phospho-eIF2α (Cell Signaling Technology #9721, Danvers, MA, USA), rabbit anti-eIF2α (Abcam# ab26197, Toronto, ON, Canada), rabbit anti-XIAP (Cell Signaling Technology #2045), rabbit anti-RIAP3 ([57]; kindly provided by Dr. Robert Korneluk, CHEO, Ottawa, ON, Canada), rabbit anti-Bcl-xL (Cell Signaling Technology, Cat#2762S), rabbit anti-4EBP1 (Cell Signaling Technology #9644), mouse anti-EIF5B (Santa-Cruz #sc-393564), rabbit anti-Maraba viral proteins, mouse anti-α-Tubulin (Abcam #ab7291), mouse anti-GAPDH (Advanced Immunochemical #2-RGM2). Probed membranes were incubated with species specific HRP-linked (anti-mouse, Cell Signaling Technology, Cat#7076S; anti-rabbit, Cell Signaling Technology, Cat#7074S) secondary antibodies. HRP signals were detected on film (GE Healthcare, Mississauga, ON, Canada) using ECL reagent (GE Healthcare). The densitometry analysis was performed using Image Studio version 5.2 software (LI-COR Biosciences, Lincoln, NE, USA).

### 4.5. [^35^S]-Methionine Labeling

Cells were washed twice in 1 mL Methionine and Cysteine free DMEM supplemented with 10% FBS and 2 mM l-Glutamine, then incubated in 1 mL DMEM-Met, Cys for 15 min at 37 °C and 5% CO_2_. For radiolabeling newly synthesized proteins, cells were pulse-labeled with 1 mL of 100 µCi ^35^S-Met, Cys (Perkin Elmer, NEG772) for 20 min at 37 °C at varying time, then washed twice with pre-chilled 1× PBS buffer and lysed in RIPA buffer (as previously described). Total protein content were separated on a 10% SDS-PAGE gel and stained with Coomassie blue dye. The signals of radiolabeled proteins were visualized on X-ray hyper-sensitive film.

### 4.6. Immunofluorescence and Confocal Microscopy

MEFs were seeded on cover slips in 6-well plates for 24 h. Time course MG1 infection was carried out after removing the culture medium from cells and replacing 2 mL of complete DMEM containing GFP-MG1 virus at MOI of 0.1 for 12 h. Cells were washed 3× in 1× PBS at 0, 3, 6, 9 and 12 hpi and fixed with 3.7% Paraformaldehyde for 20 min. Cells were permeabilized with 0.2% *v*/*v* Triton X-100 solution for 5 min, followed by blocking with FCS blocking buffer (1% FCS, 0.2% BSA, 0.4% Triton X-100 in 1× PBS buffer). Coverslips were incubated overnight at 4 °C with rabbit anti-TIAR (1:400 dilution in Triton X-100/BSA buffer, Cell Signaling Technology #D32D3). Coverslips were washed 3× for 5 min with Triton X-100/BSA buffer and incubated with secondary antibody for 1 h (Alexa Fluor anti-rabbit 594, 1:1000 dilution in Triton X-100/BSA buffer) on shaker at room temperature. Nuclei of the cells were stained using 1 μg/mL Hoechst 33342 solution (Trihydrochloride Trihydrate, Invitrogen, Carlsbad, CA, USA) in 1× PBS for 5 min. Coverslips were mounted onto glass slides using Dako Fluorescent Mounting Medium (Dako North America, Inc., Santa Clara, CA, USA). Cells were visualized using the 60× objective with water (Olympus Fluoview FV-1000 Laser Confocal Microscope, Richmond Hill, ON, Canada).

### 4.7. RNA Extraction and RT-qPCR

Total RNA was extracted using RNAzol^®^RT following the manufacturer’s protocol. The RNA yield was solubilized in RNAse-free water and the concentration was measured using Nano-drop1000. Reverse transcription was generated by using qScript^TM^ cDNA SuperMix (Quanta Bioscience, Beverly, MA, USA). Quantitative PCR was performed using SYBR green Master Mix (Qiagen, Toronto, ON, Canada) on Eppendorf qPCR machine with the following gene-specific primers: Hs-BCL2L1 (Qiagen, CAT# QT00997423), Hs-XIAP (Qiagen, CAT# QT00042854), Hs-RPL36A (Qiagen, CAT# QT01668030), Hs-RPL13A (Qiagen, CAT# QT02321333), mouse PPIA (MHK-1, RealTime Primers, Burlington, ON, Canada), mouse RPL13A (MHK-1, RealTime Primers), MG1 primers (forward 5′-GGTGATGGGCAGACTATGAAA-3, reverse 5′-CCTAAGGCCAAGAAACAAAAGAG-3).

### 4.8. Kinetic Live Cell Imaging

To determine the cytotoxicity and rate of viral infection, cells were seeded into 6-well plates for 24 h. After treating the cells with MG1 virus at MOI of 0.1 and 250 nM of Cytotox Red reagent (Essen Bioscience, Ann Arbor, MI, USA), fluorescent signals from Cytotox Red dye and GFP-MG1, as well as cell confluence were monitored in real time using IncuCyte^TM^ ZOOM Content Kinetic Imaging System (Essen Bioscience). The rate of Cytotoxicity was determined by normalizing the number of Cytotox Red positive cells to the phase confluence and the rate of MG1 infection was calculated as the percentage of GFP-expressing cells to the phase confluence.

### 4.9. Statistical Analysis

Data are represented as a mean ± standard deviation of at least three independent biological replicates. Collected data from the repeated experiments were exported to GraphPad Prism version 5.00 (GraphPad software, San Diego, CA, USA) software for graph and statistical analysis. For RT-qPCR experiments, data were represented relative to the geometric mean of RPL13A and RPL36A as human control genes and RPL13A and PPIA as mouse control genes. Student’s *t*-test and Two-way ANOVA were performed to determine the *p*-value and statistical significance.
